# A New Model to Estimate Prognosis in Patients with Hepatocellular Carcinoma after Yttrium-90 Radioembolization

**DOI:** 10.1371/journal.pone.0082225

**Published:** 2013-12-18

**Authors:** Zhihong Weng, Judith Ertle, Shaoping Zheng, Thomas Lauenstein, Stefan Mueller, Andreas Bockisch, Guido Gerken, Dongliang Yang, Joerg F. Schlaak

**Affiliations:** 1 Department of Hepatology and Gastroenterology, University Hospital of Essen, Essen, Germany; 2 Department of Infectious Disease, Union Hospital, Tongji Medical College, Huazhong University of Science and Technology, Wuhan, China; 3 Department of Ultrasonography, Union Hospital, Tongji Medical College, Huazhong University of Science and Technology, Wuhan, China; 4 Institute for Diagnostic and Interventional Radiology and Neuroradiology, University Hospital of Essen, Essen, Germany; 5 Institute for Nuclear Medicine, University Hospital of Essen, Essen, Germany; Icahn School of Medicine at Mount Sinai, United States of America

## Abstract

**Aims:**

The current prognostic model to estimate the survival in hepatocellular carcinoma (HCC) patients treated with transarterial hepatic selective internal radiotherapy (SIRT) is not fully characterized. The aim of this study was to establish a new scoring model including assessment of both tumor responses and therapy-induced systemic changes in HCC patients to predict survival at an early time point post-SIRT.

**Methods and materials:**

Between 2008 and 2012, 149 HCC patients treated with SIRT were included into this study. CT images and biomarkers in blood tested at one month post-SIRT were analyzed and correlated with clinical outcome. Tumor responses were assessed by RECIST 1.1, mRECIST, and Choi criteria. Kaplan-Meier methods were used to estimate survival curves. Cox regression was used in uni- and multivariable survival analyses and in the establishment of a prognostic model.

**Results:**

A multivariate proportional hazards model was created based on the tumor response, the number of tumor nodules, the score of the model for end stage liver disease (MELD), and the serum C-reactive protein levels which were independent predictors of survival in HCC patients at one month post-SIRT. This prognostic model accurately differentiated the outcome of patients with different risk scores in this cohort (P<0.001). The model also had the ability to assign a predicted survival probability for individual patients.

**Conclusions:**

A new model to predict survival of HCC patients mainly based on tumor responses and therapy-induced systemic changes provides reliable prognosis and accurately discriminates the survival at an early time point after SIRT in these patients.

## Introduction

Hepatocellular carcinoma (HCC) is the sixth most common malignancy worldwide with increasing incidence especially in the western world [1], [2]. The majority of HCC are diagnosed at an intermediate or advanced stage [3]. Curative treatments (resection, transplantation and radiofrequency) are only suitable for very early and early stage patients with compensated liver function. For patients who are not eligible for curative treatments with mainly intrahepatic disease, local-ablative therapies play an important role in reducing tumor burden, providing palliation of symptoms, and increasing survival [4]. Selective internal radioembolization (SIRT) with Yttrium-90 microspheres is an established local-ablative therapy [5] for intermediate and particularly advanced stages. The radiation can be selectively administrated to the intrahepatic tumors largely avoiding radiation to the normal liver parenchyma [6]. It produces initial average disease control rates above 80% and is usually very well tolerated. When compared to the standard of care for the intermediate and advanced stages, radioembolization consistently provided similar survival rates [7].

Staging systems are commonly used to classify malignant diseases. Staging of the underlying end-stage liver disease in HCC is usually performed using the Child-Pugh classification [8], or the model for end stage liver disease (MELD) [9]. However, neither of these systems includes the tumor burden which has important implications for survival. Other systems include both tumor burden and underlying liver function [10]–[12], however these systems may not have a prognostic role in non-surgical patients [13]. The Barcelona Clinic Liver Cancer (BCLC) staging system has prognostic value and links tumor stage to treatment strategy [14], [15]. However, a distinct feature of HCC is that, in addition to the tumor responses, the therapy-induced changes in liver function such as aspartate amino transferase (AST) and total bilirubin, and general condition of the patients after treatment also play a crucial role for outcome prediction [16], [17]. In addition, C-reactive protein (CRP) is found to be a prognostic factor for patients with HCC [18], [19]. More recently, the detection of tumor markers [20] or circulating tumor cells [21] has been used to assess prognosis after liver resection. A report from European Network on Radioembolization with Yttrium-90 Resin Microspheres indicated that the most significant independent prognostic factors in patients with HCC were Eastern Cooperative Oncology Group (ECOG) status, tumor burden, international normalized ratio (INR), and extrahepatic disease on baseline [22]. Current prognostic models were established based on the baseline data of HCC patients before treatment. However, there is only little information about prognostic parameters that predict the clinical outcome of HCC patients at an early time point post-SIRT.

In this study, we have analyzed possible prognostic predictors including tumor responses, therapy-induced systemic changes in a cohort of HCC patients after SIRT. Our aim was to establish a scoring model which may provide reliable prognostic information about the clinical outcome of these patients at an early time point post-therapy.

## Patients and Methods

### Patients

The records of consecutive patients who had received Yttrium-90 radioembolization therapy for HCC at the University Hospital of Essen, Germany, from June 2008 until December 2012 were reviewed. A total of 149 patients were identified and enrolled into this study. Baseline demographics and tumor characteristics are summarized in Table 1. The median age of the cohort was 68.5 years and the majority was male. Non-alcoholic steatohepatitis (NASH) was the predominant etiology of liver disease in this European cohort. Approx. half of the patients (53%) had radiological evidence of cirrhosis such as splenomegaly, portal hypertension, and ascites. The median MELD score was 8.0.

**Table 1:** Patient baseline demographics and tumor characteristics.

Variable	Value
Age (years)	68.5 [14–88]
Men	120 (81%)
Etiology of HCC	
HCV	22 (15%)
HBV	22 (15%)
HBV + HCV	12 (8%)
NASH	41 (28%)
Others	16 (10%)
Cryptogenic	36 (24%)
MELD score	8 [6]–[26]
Bilirubin (mg/dl)	0.7 [0.2–4.9]
INR	1.1 [0.9–2.8]
Creatinine (mg/dl)	1.0 [0.6–8.0]
CRP (mg/dl)	1.2 [0.1–54.0]
Albumin (g/dl)	4.0 [2.6–46.0]
Cirrhosis	79 (53%)
AFP (U/mL)	55.5 [0.8–55791.0]
Number of lesions	
1	67 (45%)
2–5	55 (37%)
> 5	27 (18%)
Size of the largest lesion	
≤3 cm	22 (15%)
3–5 cm	42 (28%)
5–10 cm	60 (40%)
> 10 cm	25 (17%)

[interquartile range] are shown. HBV, hepatitis B virus; HCV, hepatitis C virus; NASH, Non-alcoholic steatohepatitis; MELD, the model for end stage liver disease; CRP, C-reactive protein; AFP, a-fetoprotein. Number (proportion) or median

All data were analyzed retrospectively in an anonymous fashion according to the principles expressed in the Declaration of Helsinki which was approved by the Ethics Committee of the University Hospital of Essen. All patients gave their written consent that their information was stored in the hospital database and could be used for research.

### Radioembolization using ^90^Y-glass microspheres

The microspheres used were glass-based (TheraSphere, Ottawa, Canada) and are composed of 20–25 µm particles. Pretreatment mesenteric angiography and technetium-99 m macroaggregated albumin scans were performed to assess gastrointestinal flow and lung shunting [23]. Radioembolization was considered for those patients with HCC who were not eligible for curative treatments (e.g., resection, liver transplantation, local ablation) and were not considered for systemic therapy based on clinical judgment by multidisciplinary teams in University Hospital of Essen. The criteria for patient selection include fairly preserved liver function, portal vein thrombosis or extensive tumor burden (e.g., main tumor >10 cm and/or uncountable tumor nodules) and exclude patients with abnormal organ or bone marrow function or other clinical signs of liver failure on physical examination [24], [25]. All SIRT procedures were performed under angiographic control and local anaesthesia.

### Follow-up

One month after the intervention a physical examination, an abdominal CT scan and blood tests were performed. Thereafter, assessment was performed every 3 months. The endpoint of our study was December 31, 2012; the death of patient was confirmed by using the social security death index. Otherwise, patients were censored at the endpoint of our study. The CT attenuation (density) of each tumor was measured in Hounsfield units (HU) on the portal venous phase by drawing a region of interest around the margin of the entire tumor at baseline and 1-month after SIRT. The HU of all lesions were combined and a mean for each patient was calculated [26]. Tumor responses were assessed using RECIST 1.1, mRECIST, and Choi criteria [26]–[28] (Table 2).

**Table 2:** Definition of target radiological responses.

	RECIST 1.1	mRECIST	Choi criteria
Complete response(CR)	Disappearance of all target lesions	Disappearance of any intratumoral arterial enhancement in all target lesions	Disappearance of all target lesions
Partial response(PR)	At least a 30% decrease in the sum of the greatest unidimensional diameters of target lesions, taking as reference the baseline sum of the diameters of target lesions	At least a 30% decrease in the sum of unidimensional diameters of viable (enhancement in the arterial phase) target lesions, taking as reference the baseline sum of the diameters of target lesions	Decrease in tumor size ≥10% or decrease in tumor density ≥15% on CT
Stable disease(SD)	Any cases that do not qualify for either partial response or progressive disease	Any cases that do not qualify for either partial response or progressive disease	Does not meet the criteria for CR, PR, or PD
Progressive disease(PD)	An increase of at least 20% in the sum of the diameters of target lesions, taking as reference the smallest sum of the diameters of target lesions recorded since treatment started	An increase of at least 20% in the sum of the diameters of viable (enhancing) target lesions, taking as reference the smallest sum of the diameters of viable (enhancing) target lesions recorded since treatment started	Increase in tumor size ≥10% and does not meet PR criteria by tumor density

### Statistics

Survival time was calculated from the date of initial treatment to the date of death or loss to follow-up. The Kaplan–Meier analysis was applied to compare the survival distributions of different groups of patients. The MELD score was calculated using the equation: 9.57x ln [creatinine (mg/dl)] + 3.78 x ln [bilirubin (mg/dl)] + 11.2 x ln (international normalized ratio) + 6.43 [29], where the minimal values were set to 1.0 for calculation purposes.

The variables acquired at one month after the initial SIRT with a p value of <0.05 in the univariate analysis were selected to develop a multivariate Cox proportional hazards model with p value criteria for inclusion <0.1 (backward conditional). SPSS 18.0 software package was used for data analysis. The continuous variables from blood test were logarithmized and entered the survival analysis. A p value of <0.05 was considered statistically significant with a confidence interval of 95%.

## Results

### Model derivation cohort

**Figure 1:**
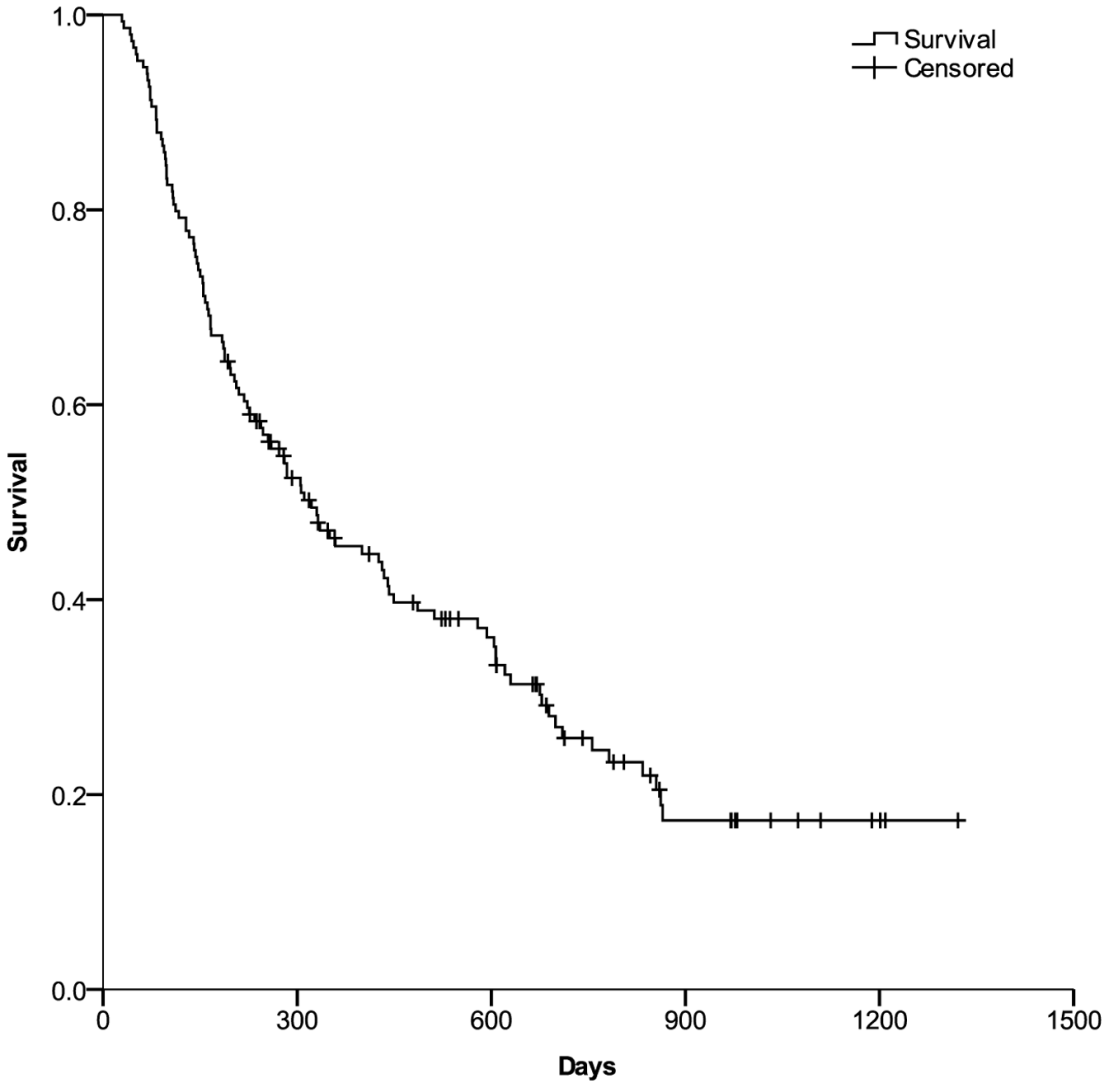
The overall survival in the whole series. Kaplan–Meier curves were generated to show the overall survival of the whole HCC patients after SIRT.

149 patients with HCC were treated with Yttrium-90 transarterial radioembolization. The most common adverse events were fatigue, nausea, vomiting, and abdominal pain. 106 (71%) patients died, 37 patients were alive, and 6 patients were lost to follow up at the endpoint of our study. As shown in Figure 1, the mean overall survival in the whole series was 502 days (95% confidence interval [CI], 425–580 days), and 1- and 2-year survival rates were 45.5% and 25.8%, respectively.

### Predictors of survival

**Figure 2:**
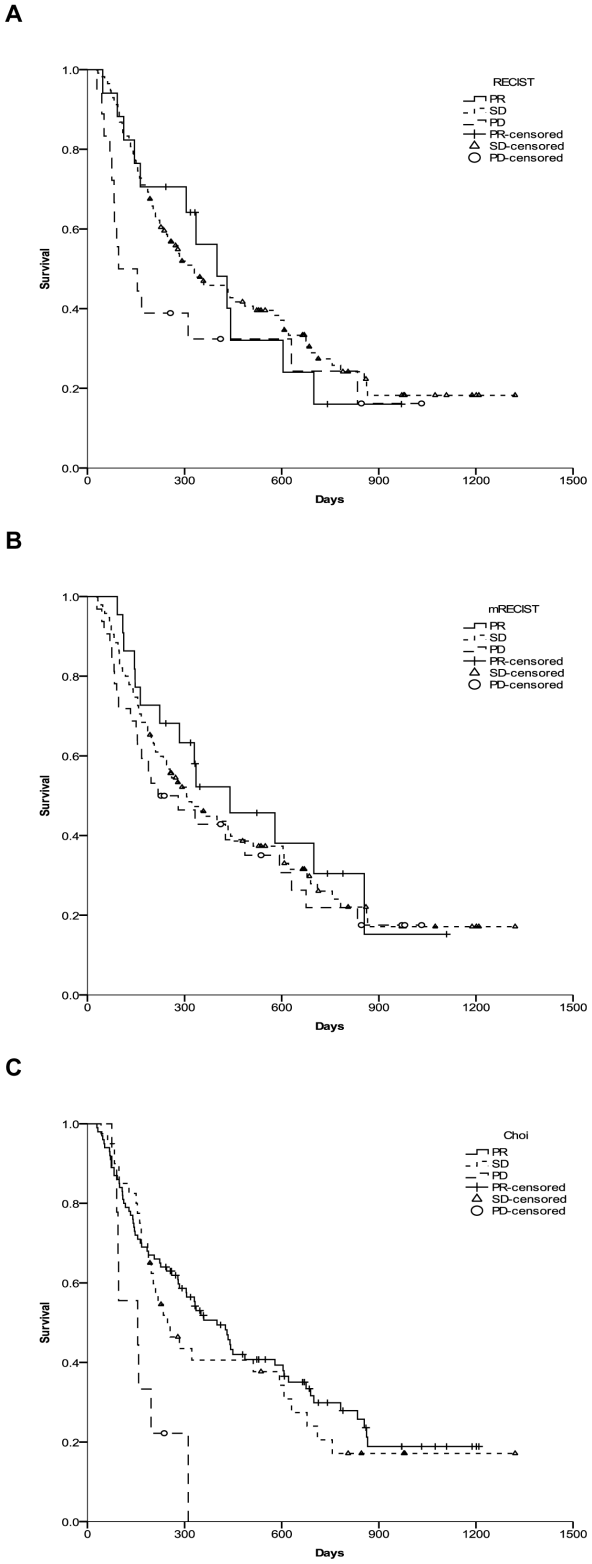
Kaplan–Meier curves were generated to compare survival between PR, SD, and PD according to three radiological assessment methods. HCC patients undergoing SIRT had radiological responses, as evaluated by three criteria: (A) RECIST 1.1, (B) mRECIST, and (C) Choi, carried out at one month post-SIRT.

Kaplan–Meier methods were used to calculate the median survival times and the survival probabilities for the “partial response (PR), stable disease (SD), and progression disease (PD)” according to RECIST 1.1, mRECIST, and Choi criteria. One month after SIRT therapy, the tumor responses for RECIST 1.1 and mRECIST were not associated with the survival in our setting (p = 0.292 and p = 0.602, respectively, Figure 2A and B). By contrast, the survival rates between PR, SD, and PD when the Choi criteria were applied were significantly different (p =  0.007, Figure 2C).

Then, we looked at the association between survival and clinical parameters including age, etiology, the largest size of tumor, the number of lesions, serum markers such as bilirubin, creatinine, INR, CRP, albumin, and AFP. In the univariate analysis, several factors evaluated at one month after SIRT showed prognostic impact concerning overall survival including the tumor response for Choi criteria, the number of nodules, MELD score, and the serum levels of bilirubin, INR, and CRP (Table 3).

**Table 3:** Univariate and Multivariate Analyses of Risk Factors for Survival.

	Univariate		Multivariate		
Variable	Hazard Ratio(95% [CI])	*P* Value	Hazard Ratio(95% [CI])	*P* Value	coefficient
Age	0.998 (0.981, 1.016)	0.861			
Gender	1.060 (0.651, 1.725)	0.815			
Etiology†	0.849 (0.565, 1.276)	0.430			
MELD	1.091 (1.048, 1.136)	<0.001	1.097 (1.054, 1.143)	<0.001	0.093
ln (Bilirubin)	2.152 (1.653, 2.802)	<0.001			
ln (Creatinine)	1.039 (0.586, 1.840)	0.897			
ln (INR)	3.017 (1.377, 6.609)	0.006			
ln (Albumin)	1.081 (0.710, 1.646)	0.716			
ln (AFP)	1.051 (0.971, 1.136)	0.217			
ln (CRP)	1.484 (1.259, 1.751)	<0.001	1.553 (1.304, 1.850)	<0.001	0.440
No. nodule††	1.454 (1.035, 2.043)	0.031	1.725 (1.240, 2.400)	0.001	0.545
Size†††	1.352 (0.927, 1.972)	0.117			
Choi ††††	1.453 (1.046, 2.018)	0.026	1.393 (0.992, 1.956)	0.050	0.331

= viral, 0 = nonviral. Etiology: 1

= 1, 2 = 2–5, 3 = 5 or greater. Number of nodules: 1

= <3 cm, 2 = 3–10 cm, 3 = >10 cm. Size: 1

= PR or CR, 2 = SD, 3 = PD. Choi response: 1

### Multivariate model

When variables with univariate significance were introduced into in a multivariate model, the tumor response for Choi criteria, MELD score, the number of lesions, and serum CRP level were selected as independent prognostic predictors of survival (Table 2). Based on the multivariate model, a risk score can be calculated using the formula: Risk Score = 0.545* (tumor number) + 0.331* (Choi response) + 0.093* (MELD score) + 0.44*ln (CRP). Further, using the means of covariate (tumor number, Choi response, MELD score and ln (CRP)) of the whole patients in this study showed by SPSS, the mean risk score 2.6 of this cohort can be calculated according to the formula. At the same time, the survival table generated by the SPSS showed the estimated survival probabilities for a patient with a risk score of 2.6 (Table 4). We defined these survival probabilities as S_0_ (t). To calculate the probability of survival at t months of a given patient use the following equation: S(t) = S_0_(t)^exp(score−2.6)^, as described previously [30].

**Table 4:** Calculation of Probability of Survival According to the Risk Score.

Months	3	6	12	18	24
S_0_ (t)	90.0%	68.7%	43.9%	34.6%	20.9%

_0_ (t) gives the estimated survival probabilities for a patient with a risk score of 2.6 which is the mean risk score of the whole patients in this study. To calculate the probability of survival at t months of a given patient use the following equation: S(t) = S_0_(t)^exp(score−2.6)^. S

**Figure 3:**
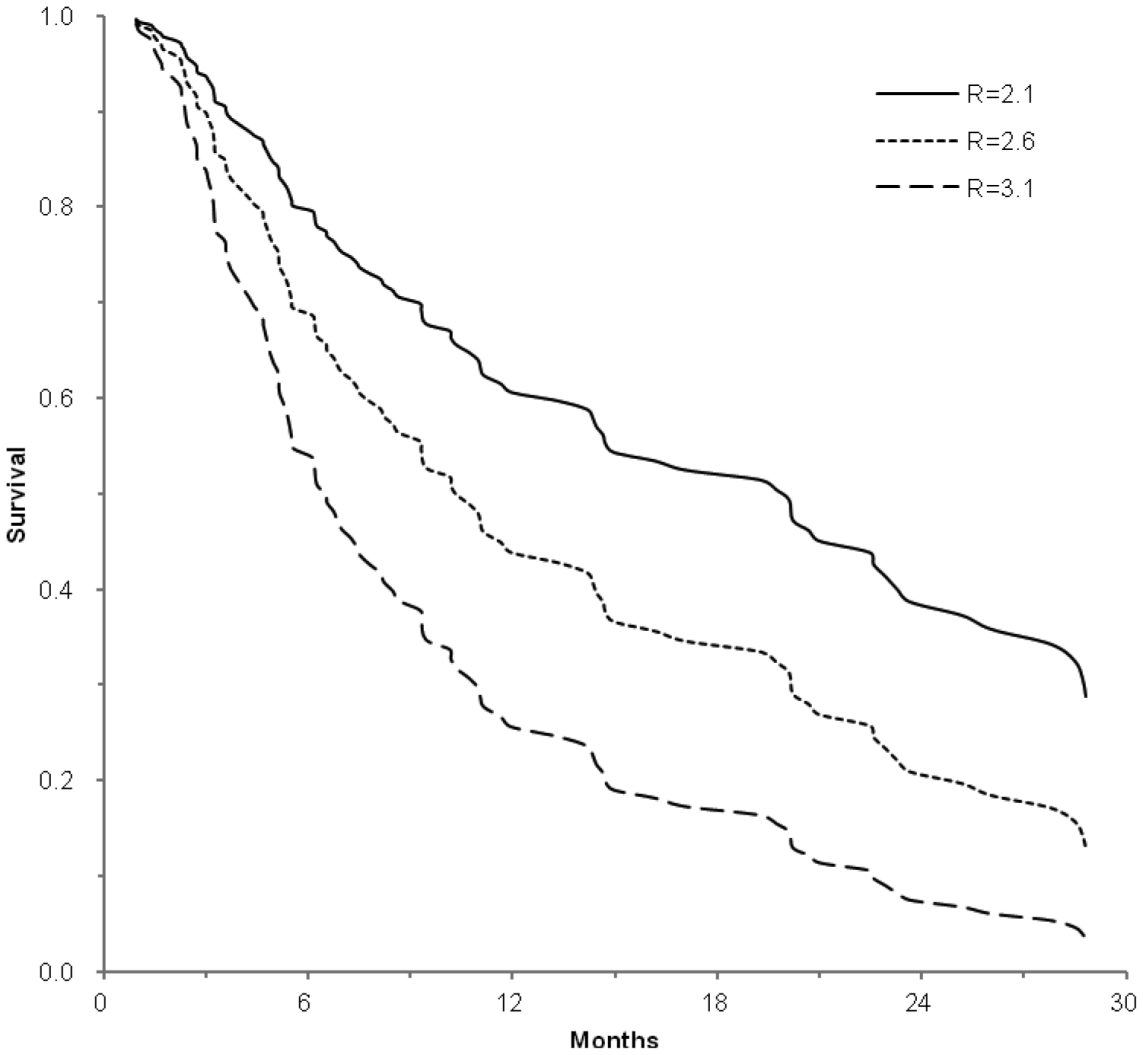
Expected survival of three hypothetical patients. Base on the equation in Table 4, the expected survival probability can be calculated in individual patients with score 2.1, 2.6, and 3.1, respectively.

The c-statistics of the model for this cohort was 0.731 (95% [CI]  =  0.643–0.818). The c-statistics is defined as probability of concordance given comparability, and a c-statistics greater than 0.7 is generally considered a useful test [31]. When being applied in individual patients, the score can calculate the expected survival probability. For example, the 1- and 2-year survival probability in patients in the lowest quartile (score ≤2.1), was 60.7% and 38.7%, respectively. While in the highest quartile (score ≥3.1), survival sharply decreased to 25.7% and 7.6% at 1 and 2 years, respectively (Figure 3).

**Figure 4:**
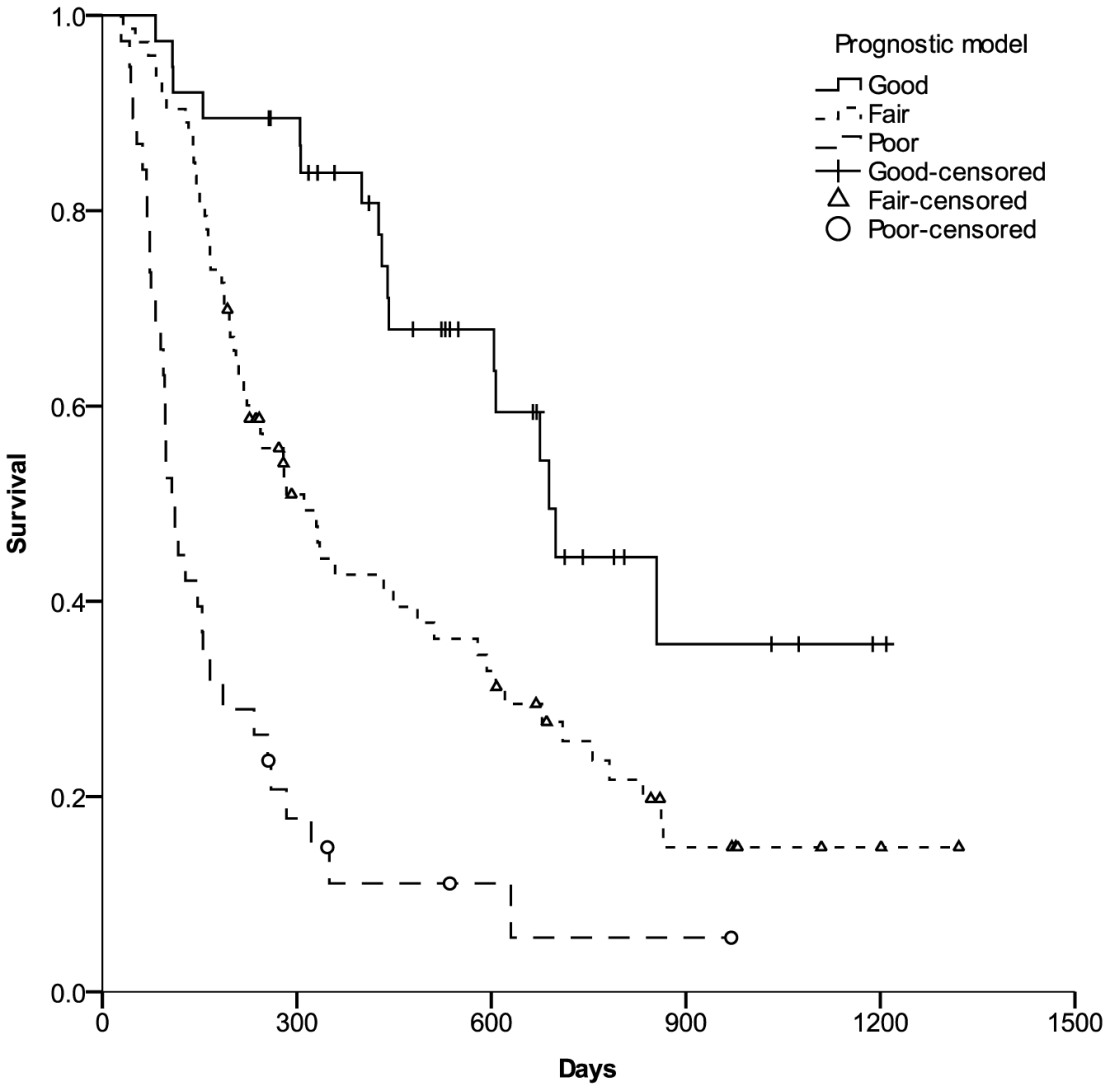
The survival probabilities among patients with good, fair and poor prognosis. Kaplan–Meier curves were generated to compare survival among patients with good, fair, and poor prognosis defined by the score according to the model at one month post-SIRT.

This model showed that patients with lower scores consistently had a better long-term survival. When the prognosis of patients with risk score less than 2.1 were defined as “Good”, patients with scores higher than 3.1 were “Poor”, and other patients with score between 2.1 and 3.1 were classified as “Fair”. There were significant differences in the mean survival times (758, 493 and 208 days, respectively, P < 0.001) and the survival probabilities among patients with good, fair, and poor prognosis as analysed by the Kaplan–Meier method (P < 0.001, Figure 4.)

## Discussion

Hepatocellular carcinoma (HCC), resulting in at least 500,000 deaths per year accounts for 90% of all liver cancers [32]. So far, some cancer staging systems, including parameters of tumor burden and severity of dysfunction, have been proposed to estimate the long-term survival of HCC patients [14], [33]. However, they were largely developed based on the baseline data of the serum markers and tumor characteristics of HCC patients before treatment. A prognostic model only based on baseline information may not be sufficient to estimate survival after interventional treatment, however. And the applicability of staging systems of HCC is also dependent on the selected treatment. In this study, we specifically investigated the prognostic predictors in a cohort of HCC patients at one month after treatment with SIRT and established a prognostic scoring model based on the following factors: the tumor response, the number of tumor lesions, MELD score and serum CRP levels. The new model accurately predicts the outcome in different strata of HCC patients after SIRT treatment.

It is well known that the determination of a prognosis is much more complex in HCC patients than in patients with other solid tumors due to the therapy-induced changes of liver function [34] as well as the tumor response to SIRT treatment. And some changes of other markers may indicate the tumor improvement without any obvious signs of tumor shrinkage or regression [35], [36]. For this reason, to establish a reliable prognostic model, it is imperative to include assessment of both tumor responses and therapy-induced changes of serum biomarkers which represent the systemic responses in HCC patients after SIRT treatment.

SIRT with 90-yttrium microspheres is an emerging tool for the treatment of primary and metastatic liver cancer that has shown promising efficiency. SIRT may lead to disease stabilization without actual shrinkage of tumor size, but showing decrease in hypervascularity and producing necrosis. The Choi criteria consider not only tumor size but also changes in tumor density on CT, resulting in a higher predictability of response [26]. Our results showed that Choi response accurately discriminated the outcome of HCC patients after SIRT treatment, while mRECIST response which was regarded as an independent prognostic factor for survival in HCC patients treated with transarterial chemoembolization [37] had no prognostic value in the cohort studied, neither had RECIST 1.1.

In our study the underlying liver function as assessed by the MELD score, could also serve as an independent predictor of survival which was consistent with previous data [14]. The MELD score consists of laboratory variables that are widely available and reproducible. CRP is known as a sensitive but non-specific inflammatory marker produced by the liver which is associated with tumor progression in patients with HCC [18] and is a useful biomarker for predicting outcomes after liver transplantation in patients with HCC [19]. Here, high serum CRP levels had an unfavorable prognostic relevance.

We incorporated Choi criteria as a gauge of tumor response and MELD score as an indicator of liver disease severity. The multivariate analysis showed that the tumor response, the number of tumor nodules, MELD score and serum CRP levels independently determine the survival of these patients. A prognostic scoring model was established according to these variables. Being a continuous score, the new model could accurately differentiate the outcome of patients with different risk scores. High-risk patients who are expected to have a decreased survival probability can be thus identified at only one month after SIRT and could be subjected to intensify clinical monitoring or could be considered for early alternative therapeutic strategies. Specifically, the model has the ability to assign predicted survival probabilities. It is easy to calculate the risk score according to the model equation.

Although the number of patients included in this study is limited, concerning the evaluation of prognosis after this locoregional therapy, the assessment of both tumor responses and therapy-induced changes of serum biomarkers which represent the systemic responses would be more relevant for HCC patients. In conclusion, this retrospective single-center study on a homogenous HCC cohort treated by SIRT is the first exploring approach to identify tumor responses and therapy-related changes of serum markers at one month after treatment for the early estimation of prognosis which is needed to be validated by further, larger prospective treatment studies.
